# How driving endonuclease genes can be used to combat pests and disease vectors

**DOI:** 10.1186/s12915-017-0420-4

**Published:** 2017-09-11

**Authors:** H. Charles J. Godfray, Ace North, Austin Burt

**Affiliations:** 1 Department of Zoology, University of Oxford, South Parks Road, Oxford, OX1 3PS UK; 2 Department of Life Sciences, Imperial College London, Silwood Park, Ascot, Berkshire SL5 7PY UK

**Keywords:** Gene drive, Gene editing, Endonucleases, CRISPR-Cas9, Vector control, Pest control, Mosquitoes

## Abstract

Driving endonuclease genes (DEGs) spread through a population by a non-Mendelian mechanism. In a heterozygote, the protein encoded by a DEG causes a double-strand break in the homologous chromosome opposite to where its gene is inserted and when the break is repaired using the homologue as a template the DEG heterozygote is converted to a homozygote. Some DEGs occur naturally while several classes of endonucleases can be engineered to spread in this way, with CRISPR-Cas9 based systems being particularly flexible. There is great interest in using driving endonuclease genes to impose a genetic load on insects that vector diseases or are economic pests to reduce their population density, or to introduce a beneficial gene such as one that might interrupt disease transmission. This paper reviews both the population genetics and population dynamics of DEGs. It summarises the theory that guides the design of DEG constructs intended to perform different functions. It also reviews the studies that have explored the likelihood of resistance to DEG phenotypes arising, and how this risk may be reduced. The review is intended for a general audience and mathematical details are kept to a minimum.

**Electronic supplementary material:**

The online version of this article (doi:10.1186/s12915-017-0420-4) contains supplementary material, which is available to authorized users.

## What gene drive is

In the absence of selection a particular gene on a particular chromosome has a 50:50 chance of being present in an individual gamete. Gene drive occurs when a gene is able in some way to subvert Mendelian segregation so that it is overrepresented in the gametes. There is currently great interest in the possibility of using genetic technologies to drive genes through populations of harmful insect pests or vectors [1–8]. The aim of some interventions is to reduce insect densities (population suppression) while the purpose of others is to bring about a genetic change (population replacement) so that, for example, an insect is no longer capable of transmitting a disease. Ideas about utilising gene drive in pest and vector management date back to the 1960s when Hickey and Craig [9, 10] and Curtis [11] explored the possibility of transforming mosquito populations using self-spreading chromosomal variants, and when the influential evolutionary biologist WD Hamilton [12] presented models showing that genes that spread through non-Mendelian mechanisms may cause population suppression or elimination.

Many examples of natural gene drive were discovered and investigated in the 20th century [13], and sporadic discussion about applied gene drive continued in the evolutionary and applied biology literatures, but serious work on operationalizing the technology awaited the new century. A strong stimulus was provided by the discovery of homing endonuclease genes (HEGs) in single-celled eukaryotes [14], and the suggestion by Burt [15] that they might be used to control the major vectors of malaria and other human diseases. More recently, the discovery of the CRISPR-Cas9 system in bacteria [16, 17] and the realisation that it may be adapted to create an artificial drive mechanism in eukaryotes analogous to HEGs (zinc-finger nucleases and TALENs might similarly be employed) [3, 18] has brought many new laboratories into the field (see also Additional file 1: Note 1). At the same time the unexpected emergence of the Zika virus in South America [19] has underscored the relatively restricted armoury we have to defend ourselves against vector-borne diseases and stimulated work on novel approaches.

Gene drive works through competition amongst alleles at the level of the gene rather than the individual (hence they are often called ‘selfish genes’). That it works is demonstrated by mathematical models [15, 20] as well as by observations of nature [21] and, increasingly, proof of principle in the laboratory [22–24]. However, the logic behind the spread can be counterintuitive and alien to biologists familiar with thinking of selection acting primarily through the differential survival and reproduction of individuals, while the technical literature is difficult to access without a modelling background. The aim of this review is to summarise the theoretical population biology relevant to the application of gene drive using endonucleases for pest and vector control. While providing an entry into the modelling literature, we aim to do this without anything but the minimum of mathematics, relying chiefly on intuitive and graphical arguments. We concentrate on what we call driving endonuclease genes (DEGs), which include HEGs, modified CRISPR-Cas9 systems and other existing and likely-to-be-discovered constructs that spread in the same general way, and we chiefly focus on population suppression and replacement (see Additional file 1: Note 2 for a further way of using gene drive). We do not discuss in any detail the molecular biology of DEGs (for reviews see [3, 4, 13]) or non-DEG drive mechanisms [1, 4]. Finally, we focus on applications of DEGs to address problems arising from insect pests and vectors, the group that has attracted most attention so far, although nearly all the concepts explored here apply equally to gene drive in other sexually reproducing organisms.

## Why driving endonuclease genes spread

**Fig. 1.**
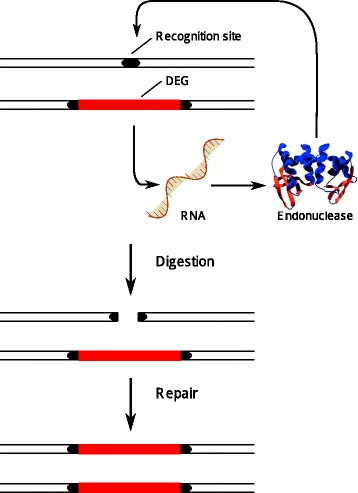
Driving endonuclease genes (DEGs) code for an endonuclease that targets a recognition site where it causes a double-strand break. The DEG is inserted in the chromosome in the same position where the recognition site occurs in the wild type. In a heterozygote, the DNA break is repaired using the homologue, leading to a DEG homozygote (after [13])

The common feature that defines a DEG is its ability to be copied from one chromosome to another such that a ‘heterozygote’ (a diploid individual with the gene on one but not the other chromosome) becomes a ‘homozygote’. It does this by coding for an endonuclease that causes a double-strand break in the same location on the homologous chromosome. Because the DEG is inserted in the middle of its own recognition site, only the chromosome not containing the gene is cut. The cell (normally) repairs the double-strand break using the chromosome carrying the DEG as a template (homology-directed repair) and hence the gene is copied to the damaged chromosome, a process known as *homing* (Fig. 1). DEGs can also be adapted to spread through favouring one sex chromosome over another, a mechanism we return to in the section on Y Drive below.

The normal intuition that a gene spreads because it provides some advantage to the individual that carries it (typically more offspring) fails in the case of non-Mendelian genes such as DEGs, which break the normal rules of inheritance. One now has to think of the gene in competition with its alternative ‘allele’—the homologous stretch of the chromosome that does not carry a DEG. A DEG will spread when rare if an arbitrarily chosen copy of the DEG produces more copies of itself than an arbitrarily chosen example of the alternative allele [15]. Because the DEG is initially rare, it will nearly always be in a heterozygote and the fraction of offspring carrying the DEG will be ½(1 + *e*), where *e* is the so-called homing frequency. The DEG gets into half the offspring by simple Mendelian inheritance and into a fraction *e* of the rest of the offspring by the ‘homing’ action of the endonuclease. A randomly chosen example of the alternative allele will nearly always be in a homozygote (because the DEG is rare) and be transmitted through Mendelian inheritance to ½ the offspring. Because ½(1 + *e*) is greater than ½, a DEG that has no deleterious effect on its host will *always* spread, and will eventually become fixed (it excludes the other allele and achieves a frequency of 1).

Natural examples of DEGs tend to be found in self-splicing introns or inteins and probably have few effects on their host’s phenotype [25]. Values of the homing frequency (*e*) can approach 1 in these systems and this provides an extraordinarily strong selective advantage for the DEG allele. It rapidly goes to fixation where, in the absence of any further selection, it would accumulate mutations and become non-functional. DEGs can only persist in nature by jumping between species, which is probably why they are only found in unicellular organisms with a relatively unprotected germline [21, 26]. The aim of population replacement strategies is to use artificially engineered DEGs to drive a useful construct through a wild population, for example a sequence coding for a peptide that interferes with disease transmission. In the ideal case, this would have no fitness consequences for the host, and it would spread rapidly to fixation.

Population suppression strategies seek to reduce the density of a pest or vector, possibly to a level when the population can no longer sustain itself (population elimination). Unlike population replacement where ideally the DEG does not affect fitness, now a DEG is designed that does cause substantial harm to the population. Consider the case where the DEG is inserted in a chromosome in the middle of a functional gene whose activity it disrupts. Focus first on a fully dominant gene where functional homozygotes and heterozygotes have the same fitness (which we can define to be 1) but non-functional homozygotes have fitness 1 – *s*. Will the DEG spread? Let us suppose that the DEG acts after the functional gene is expressed so that the conversion of a heterozygote to a non-functional homozygote does not affect the fitness of the individual in which it occurs. When rare, the DEG is nearly always in a heterozygote and its fitness is near the wild type because the functional gene is fully dominant. It will thus begin to spread through the population for the reasons described above.

As the DEG advances through the population more and more non-functional homozygotes will be produced and this will clearly act to slow its spread. We can ask whether the DEG reaches an equilibrium frequency which we denote *q*. At equilibrium, by definition, the number of copies produced by arbitrarily chosen individual DEG and wild-type alleles should be equal—if this were not true then the frequency would change. An exactly equivalent condition is that the net costs and benefits for each allele due to the segregating DEG should be the same at the equilibrium frequency *q* (Additional file 1: Note 3). Consider first a wild-type allele: as a homozygote it is unaffected by the DEG but with probability *q* it is in a heterozygote and suffers the risk of conversion *e* (so the net effect is –*qe*). Now the DEG will be in a heterozygote with probability (1 – *q*) and gain the benefit of conversion with probability *e*, while with probability *q* it will be in a homozygote and suffer fitness costs *s* (so the net effect is (1 − *q*)*e* – *qs*). Equating the net effects for the two alleles and solving for *q* we obtain *q* = *e*/*s*.

**Fig. 2.**
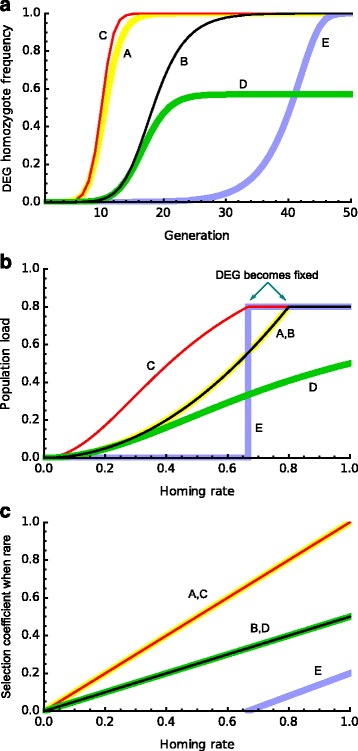
The spread of different types of DEGs where homozygote fitness is reduced by 80% (in all examples homing occurs after the gene is expressed). In all panels *A–D* are recessive DEGs: *A*, cost is increased mortality in both sexes; *B*, cost is a reduction in female fecundity and homing only in females; *C*, as *B* but homing in both sexes; *D*, as *B* but homing in males only; *E*, as *A* but now heterozygote fitness half that of homozygote. **a** The spread of different constructs when the homing rate is 0.9 and the initial frequency of the DEG is 0.01. **b** Equilibrium population load as a function of homing rate. **c** Rate of spread from rare as a function of homing rate (increase in frequency per generation)

The fate of the DEG thus depends solely and simply on homing frequency *e* and the fitness reduction *s* (note, *s* will be high for population suppression and low for population replacement). When the fitness effects are greater than the homing frequency (*s* > e) there will be a (stable) polymorphism with both alleles present but when the reverse is true (*s* < e, implying *q* > 1, which is impossible) the DEG will increase to fixation and the wild-type allele disappear (Fig. 2).

The fact that a gene that reduces population fitness can spread is the basis for the strategy of population suppression. In this simple case of a dominant functional gene it is straightforward to calculate the genetic load the spread of the DEG places on the population [20]. At equilibrium, the frequency of homozygote DEGs is *q* ^2^: relative population fitness is thus reduced from 1 to 1 – *q* ^2^ *s*. In the case where the DEG goes to fixation (*s* ≤ *e*) and so *q* = 1 then population fitness is 1 – *s*. The load is thus the same as the fitness reduction of the DEG homozygote. Where there is a polymorphism (and *q* = *e*/*s*) the load is *q* ^2^ *s* = *e* ^2^/*s*. The potential power of a potent DEG to reduce population fitness is thus very great. For example, were homing to be absolute (*e* = 1) then DEGs targeting genes that reduced population fitness to near zero (*s* → 1) would spread, leading to certain elimination.

We have dwelt at some length on this simplest of cases to try to provide an intuitive understanding of why DEGs spread. It is straightforward to relax the simplifying assumptions, though typically mathematical reasoning has to replace verbal arguments, and in Additional file (and for full details see [20]) we explore some of these issues. Potential candidate genes to be targets for DEGs include viability or fecundity genes that may be active in both sexes or just one. Theory suggests female fecundity genes may be the most effective single genes to target (Fig. 2; Additional file 1: Note 4). DEGs can still spread if they target genes where heterozygote individuals have reduced fitness, and mild heterozygote and homozygote costs are not a barrier for population replacement strategies. High heterozygote costs can lead to complex dynamic behaviours [20, 27, 28] and such genes are poorer targets when population replacement is the goal (Fig. 2; Additional file 1: Note 5). Finally, for population suppression there is a clear advantage in choosing a DEG that homes after the developmental stage during which the target gene is expressed so that the costs imposed by the DEG do not reduce the boost it gets from converting heterozygotes to homozygotes (Additional file 1: Note 6). This advantage is much less important for population replacement strategies and several recent models motivated by this type of intervention assume homing occurs early in development [28, 29].

It is also possible to explore the speed with which a DEG spreads through a population, an important question in designing deployment strategies (Additional file 1: Note 7). In Fig. 2a at the time required for a selection of DEGs to spread is plotted, which shows that rapid increases in frequency can occur over a relatively small number of generations.

Where population suppression is being attempted, multiple DEGs targeting different essential genes can be introduced into a population at the same time. The dynamics of these DEGs is largely independent (homing acts to break up linkage associations between alleles even if they are quite close together on the chromosome). Where each DEG reduces population fitness by an amount *L* (the load) the overall reduction is 1 – (1 – *L*_1_) (1 – *L*_2_) … (1 – *L*_*n*_), where the subscript indexes the load of each of the *n* DEGs. Thus, even if any individual load is quite small, the combination of multiple drags on population fitness quickly adds up [30, 31].

## Population dynamics

Population genetic arguments can tell you the extent to which a DEG can reduce average population fitness (the genetic load, *L*, expressed as a fraction between 0 and 1). But for population suppression we need to know what this means for population densities and in particular whether the load is sufficient to cause population elimination, if this is the goal. Answering this question requires an understanding of the target insect’s ecology in the field, often a more challenging task than measuring genetic parameters in the laboratory.

### Population elimination

Begin by assuming the insect lives in a constant environment and the DEG causes a substantial reduction in density such that the species is no longer subject to factors such as competition for resources that limit its density when common. Further, assume that the insect has discrete generations and that its capacity to increase when rare is *R*_*m*_, which is measured by the number of female offspring produced per female parent (the argument for insects with overlapping generations is essentially the same). The species will persist if, on average, every female more than replaces itself: *R*_*m*_ >1. In the simplest case the genetic load can be measured by the reduction in the number of female offspring produced. Thus, once the DEG is established the growth rate of the population is *R*_*m*_ (1 − *L*) and for elimination to occur *L* > 1 – 1/*R*_*m*_ [30].

A number of conclusions follow immediately from this simple calculation. First, it will be much harder to eliminate a species with a great capacity to increase when rare. Second, population reduction may work best in peripheral and marginal populations where by definition the species has a lower *R*_*m*_: thus, one might get population elimination in these areas but suppression with persistence in the core of the range. Third, any other control measure that reduced *R*_*m*_ at the same time as the DEG would increase the chance of elimination; provided the control measure does not differentially target individuals carrying the new construct, they should help and not hinder control. Fourth, where the load is not large enough to lead to inevitable elimination, the population may be so small that it is subject to demographic stochastic elimination—for example by ‘good’ luck all individuals may fail to breed and so the population is eliminated.

It is surprising how little we know about the magnitude of *R*_*m*_, even for medically and economically important insects. For the main mosquito vector of malaria in Africa (the *Anopheles gambiae* complex), Deredec et al. [30] found only one good estimate (from the famous Garki project [32] in the early 1970s in Nigeria) where *R*_*m*_ ~ 9. This suggests that a load of about 0.9 (a 90% reduction in fitness) would be required for the elimination of a population with similar ecology, though *R*_*m*_ will certainly vary across sites.

Of course, vectors and pests are not found in constant environments where *R*_*m*_ remains constant throughout the year. However, the above arguments still hold, to a good approximation, if *R*_*m*_ is replaced by its geometric (rather than arithmetic) mean over time (Additional file 1: Note 8) [33]. For some species there may be a minimum population density below which elimination always occurs (an Allee effect [34]; Additional file 1: Note 9), which will tend to increase the likelihood of population elimination.

### Population suppression

A DEG that substantially reduces population density, but does not cause elimination, would still play a valuable role in reducing the damage done by a pest or vector. However, it is hard to say how the DEG will affect population size because this requires an understanding of how it interacts with all the other ecological factors determining population size and, in particular, those that are density dependent—that is, they vary in magnitude as population density changes. Density-dependent factors are of particular importance because they determine the typical abundance of a species. The relative position in the insect’s life cycle where density dependence acts and where the organism suffers from the presence of the DEG can be very important in determining the effectiveness of the genetic intervention.

**Fig. 3.**
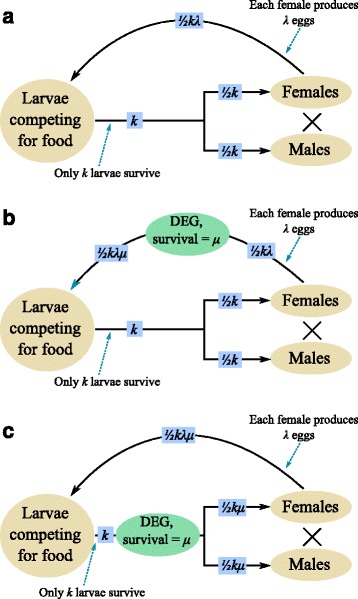
Schematic insect life cycle illustrating the significance of the relative timing of the fitness effect of the DEG and the action of density dependence. *Brown ovals* represent life cycle stages and *blue squares* population densities. **a** The absence of a DEG; **b** DEG expressed before density dependence; **c** DEG expressed after density dependence

To explore this further consider a highly idealised population with discrete generations (Fig. 3a). We assume that the population is limited by larval food supply so that exactly *k* larvae survive the juvenile period to go on to become adults irrespective of how many eggs are laid. This form of larval competition is density dependent because the *per capita* mortality risk increases with density. Thus, if 20*k* eggs hatch the risk of mortality is 19/20 but if 100*k* eggs hatch the risk of mortality is 99/100. Now assume that a DEG is present in the population at an equilibrium frequency determined by the genetic arguments described above but that the load is not sufficient to cause elimination. If the DEG acts early in the life cycle (say at the egg stage) so that only a fraction *μ* survive, then if female fecundity is large the number of eggs hatching is still likely to exceed *k* and the same number of pupae will arise. In effect what has happened is that the density-dependent mortality has reduced in severity to compensate for the fewer eggs hatching (Fig. 3b) so that the number of adults remains the same. But if the DEG acts after density dependence (Fig. 3c) the number of adults is reduced to the same proportion as the fraction surviving, *μ*.

The important point here is that it matters where in the lifecycle the DEG acts relative to where density-dependent mortality occurs and the life stage that causes the damage. To get maximum effect, the DEG should act after density dependence but before the damaging stage, otherwise the density-dependent mortality will compensate for some of the load imposed by the DEG. In the major mosquito vectors of human disease it is generally thought that density dependence occurs in the larval stage [35–37] and obviously it is the adults that transmit disease. A DEG acting in the pupal stage or immediately after the adult emerges would be optimal. A pest insect that causes damage in an early instar and where density dependence occurs later in the lifecycle might be best targeted by a DEG that acted at the egg stage.

The density-dependent processes that determine typical population abundances may operate by affecting mortality (as in the example above) or fecundity—perhaps females in high density populations have insufficient food to produce a full complement of eggs, or grow up small and stunted with reduced fecundity. Density-dependent effects may also increase generation time and so slow population growth rates. The precise shape of the density-dependent mortality function can also have important consequences for population dynamic behaviour [38] (Additional file 1: Note 10). The rather artificial example described above where exactly *k* individuals emerge from a life stage is termed perfectly compensating density dependence, but more typically the effects of density operate more smoothly without such a sharp threshold (under-compensation). Over-compensation is said to occur when low population densities in one generation lead to very large population densities in the next generation and vice versa. Were such dynamics to characterise mosquito populations then there would be concern that introducing a DEG that did not cause elimination might under some circumstances actually increase equilibrium populations [39], but what we know about these insects’ biology suggests that this is just a theoretical curiosity. Finally, studies of real populations have to take into account that both density-dependent and density-independent factors vary over time and space. Implementing population suppression successfully will thus require careful study of the population ecology of the target species.

### Species interactions

Eliminating or reducing the density of a target species will affect the dynamics of its prey, predator and competitors, and possibly the further species with which they interact. There has been little theoretical exploration of this subject to date in a specific DEG context, though community ecology provides a rich tool box to explore this question (Additional file 1: Note 11).

### Disease transmission

Many of the possible targets of DEGs are of concern because of the human, animal or plant diseases they transmit, rather than the direct harm they themselves inflict. Using DEGs to drive a gene that interferes with disease transmission through a vector population is a major goal of population replacement strategies, while population suppression aims to target the disease by lowering vector numbers rather than competence. In exactly the same way that the insect population is eliminated if a load is imposed so that each adult female has, on average, less than one female offspring, so the disease is eliminated if an initial infection leads to less than one secondary infection when the pathogen is uncommon. The number of secondary infections when the disease is rare is called the basic epidemiological number or *R*_0_ [40].

Most expressions that have been derived for the *R*_0_ of vector-borne diseases [41, 42] are the product of (i) biting insects per host and (ii) a parameter combination that includes propensity to bite, adult insect lifespan, and transmission efficiency (see Additional file 1: Note 12 for a worked example). Thus, any vector population suppression will lead to a proportionate reduction in *R*_0_ through the first component, while population replacement strategies affect *R*_0_ through the second. Obviously, vector elimination will lead to disease elimination (in the absence of alternative vector species), but are there circumstances when fully competent vectors persist (*R*_*m*_ in the presence of the DEG > 1) but their abundance is so reduced that the disease goes extinct (*R*_0_ < 1)? In a model motivated by the mosquitoes that transmit malaria, Deredec et al. [30] found circumstances where this might occur (high *R*_*m*_ but low *R*_0_), though further work including such factors as seasonality and other interventions in the model is needed to assess its relevance to disease control.

For an insect vector to transmit disease it must live long enough to bite a host and acquire the pathogen, for the pathogen to develop to a life stage in which it can be transmitted, and then for the vector to bite a second host and transmission to occur [42]. Given the high rates of daily mortality suffered by most small invertebrates, it is likely that only a relatively small fraction of long-lived individuals is responsible for disease transmission. A DEG (or other) intervention that reduced adult vector longevity might be more effective in reducing disease incidence than its effects on vector numbers might suggest [43–45]. A DEG that was expressed and caused mortality exclusively in old individuals could be particularly effective, not only because it would kill the dangerous fraction of the population but also because if the numbers involved were relatively small then natural selection for resistance (see next section) would be lower [30].

One of the consequences of reduced population size and relaxed competition is that larvae may experience reduced competition for food and emerge as larger adults. If larger adults live longer then they could be more efficient at transmitting the disease (the opposite of the process described in the last paragraph), reducing or conceivably reversing the effect of the intervention [46]. However, the situation is more complicated as smaller females may feed more often so that they may be more efficient vectors than larger individuals. Whether this occurs depends critically on the shape of the relevant density-dependent functions. More work is needed but our (unpublished) modelling suggests that quite extreme forms of prolonged lifespan are required for the size-longevity effect to counter the advantages of fewer vectors.

## Resistance and recall

Any DEG that reduces the fitness of its host will face the potential evolution of resistance. In this, DEGs are no different from any other intervention that seeks to kill or impair harmful vectors and hosts.

Perhaps the most likely type of resistance allele to arise is one that does not contain the DEG recognition site. Such an allele might be present undetected at very low frequency or may arise de novo after the DEG has been released, either due to background mutation or through the action of homing itself. Homing works because double-strand breaks are usually repaired using the homologous chromosome as a template. But repairs can also be made by end joining (EJ), which as the name suggests involves direct ligation of sundered chromosome ends [47]. If EJ leads to a perfect repair in which the DEG recognition site is completely reconstructed then there is no lasting affect and EJ can be modelled simply as a reduction in homing frequency. But if EJ destroys the DEG recognition site then an allele resistant to the DEG will have been created. Irrespective of origin, the fate of such an allele will depend on its fitness relative to the different DEG genotypes [20]. Other types of resistance are possible; for example, any mutation not in the DEG that resulted in the disabling or blocking of the DEG could be favoured. Resistance mutations generated by EJ, including non-homologous and micro-homology-mediated EJ [48], have been observed in the laboratory [23].

**Fig. 4.**
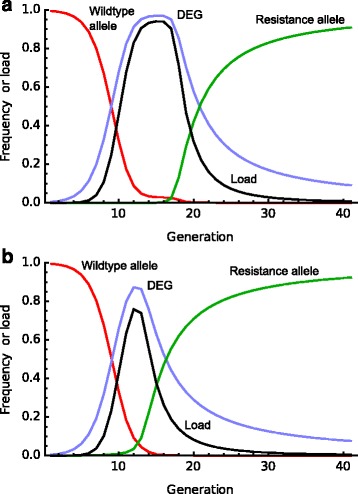
Dynamics of a resistance (or recall) allele. A DEG allele (homozygote females are sterile; homing rate =0.9) is introduced at a frequency of 1% in generation 1 and quickly spreads, imposing a population load. In **a** the DEG goes to fixation; we assume resistance alleles arise from EJ (one per thousand cleavage events) and such an allele quickly spreads, reducing the population load, initially rapidly and then more slowly. The dynamics are identical if instead of a resistance allele an artificially created recall allele is introduced. In **b** we assume that a resistance allele with the same parameters exists as a rare variant in the population at the time of DEG introduction (initial frequency 10^−6^). The allele spreads rapidly and the population experiences only a transient genetic load

The worst-case scenario for population suppression is a mutation that completely restores wild-type fitness. Consider a DEG that has gone nearly to fixation leading to substantial population suppression (but not elimination). An escape mutant that arises can spread very fast (Fig. 4) with population fitness rebounding to 95% in roughly the number of generations that it took the DEG to spread. Specifically, the number of generations scales approximately with the inverse of the log of population size and mutation rate: resistance negates the effect of the DEG more slowly when mutation rates are lower and population size larger but a tenfold difference in either leads to just a halving in the rate of fitness restoration. If standing variation for resistance is already present in a population when the DEG is deployed then it very quickly becomes selected and the intervention causes only a temporary reduction in pest or vector density (Fig. 4). The dynamics of resistance mutations are more complicated if they do not fully restore wild-type fitness and are explored in Additional file 1: Note 13.

These considerations suggest a series of strategies to try to avoid resistance. The probability of functional escape mutations can be reduced by ensuring that the DEG recognition site is in a conserved region of the gene where any change to the recognition site also destroys gene function. Third-base pair redundancy of course makes this difficult, but not impossible—in mosquitoes some sequences are, for unclear reasons, conserved at the nucleotide level for tens of millions of years [49]. A critical issue is to ensure that no alleles immune to the DEG are already segregating in the target population. Genetic surveys can detect such alleles if they are at high frequency but will be much less likely to find rare variants. Again, choosing recognition sites in conserved regions of genes is likely to be the best precautionary strategy.

A further way to avoid resistance is to combine multiple DEGs in a single intervention, or to use DEGs that cause multiple double-strand chromosome breaks [15, 29, 31]. As in combination drug therapy, the probability of resistance can be reduced by requiring multiple resistance mutations to occur simultaneously. Additional file 1: Note 14 describes an elegant analysis by Marshall et al. [31] suggesting that resistance to single CRISPR-like DEGs is almost inevitable (if it has no fitness cost), while resistance to multiple DEGs becomes exponentially more difficult as their numbers increase. Two aspects of DEGs facilitate this approach. First DEGs, especially those based on CRISPR-Cas9, can be reprogrammed to target multiple sites [3]. Such a strategy would fail if a mutation arose that suppressed all CRISPR activity, but then the second advantage, the availability of very different DEG systems (or CRISPR variants) that are unlikely to be suppressed by the same mutation, comes into play. A mutation that suppressed chromosome repair using the homologous chromosome could affect all DEG approaches though likely at the cost of reducing the organism’s overall fitness. Even then, there are other ways to use DEGs that might be ‘stacked’ to reduce the likelihood of resistance.

In the context of population replacement, Unckless et al. [28] develop a valuable theoretical framework to explore the dynamics of resistance alleles that are already present at low frequencies in a target population or that arise de novo after deployment. They assume that individuals in which homing occurs experience the fitness costs of the homozygote DEG (that is, homing occurs before expression) but that these costs are much lower than for a population suppression strategy. For replacement strategies, minimising fitness costs and targeting conserved regions for recognition sites are both important mitigation strategies. Noble et al. [29] (Additional file 1: Note 15) model a neat variant of a replacement strategy originally suggested by Esvelt et al. [3]. In their scheme, part of the payload of the construct is a copy of the gene being targeted that has been recoded at the DNA sequence level so that it is immune to cleavage but still produces a viable protein product. Any fitness cost due to recognition site disruption would thus be avoided, reducing selection for resistance. Finally, when population replacement seeks to render vector individuals incapable of transmitting a pathogen, the possibility of the latter evolving resistance to the intervention must be considered.

The strength of selection for resistance means that any artificially created resistance allele would quickly spread through the population. It would thus be possible to use natural selection to reverse the effect of any costly DEG should it be desired [15]. Such a remediation strategy might be requested by regulators, or could be used to reinstate an insect vector population after the disease it carries has gone extinct. The reconstituted population might not be genetically identical to the original wild type, but the differences could be minimised to the small number of bases in the recognition sequence. Related to this, population replacement might be achieved via a population suppression intermediate step [50, 51]. The idea here is first to impose a load on the population and then to introduce an artificial resistance allele in combination with a beneficial construct. A reason for doing this is that the end result would be the establishment of the desired construct but with the loss of the genetic element capable of drive.

Even when a DEG used for population replacement has no effect on fitness, a DEG could in principle be engineered to reinstate an allele that had been disrupted by a previous gene drive. To do this it would be necessary to prevent the original DEG from cutting the replacement allele, for example by altering the DNA sequence in a way that did not affect the protein sequence, or in a two stage process in which a sequence immune to cutting is first driven through a population followed by the desired allele once the original DEG has been lost [3]. Alternatively, Wu et al. [52] suggest that a CRISPR-Cas9 DEG system may be recalled by introducing a guide RNA that targets the Cas9 gene itself (they call this mechanism CATCHA after Cas9-triggered chain ablation [of Cas9]; Additional file 1: Note 16; Additional file 2).

These elaborations on the basic theme of DEG-drive are conceptually fascinating and potentially important. Nevertheless, the reality is that regulators will almost certainly need to be convinced that ‘basic’ gene drive is efficient and safe before they countenance more complicated constructs.

## Y Drive

There is a second way that a driving endonuclease genes can spread [15]. In species where there are differentiated sex chromosomes the two different chromosomes in the heterogametic sex can be thought of as competing to be represented amongst the gametes. Consider a DEG that is carried by the Y chromosome but which codes for an endonuclease that targets a sequence on the X chromosome. We assume males are the heterogametic sex (XY). If the double-strand break the endonuclease causes on the X is not repaired then by destroying competing gametes it gives the Y chromosome on which it is carried an advantage. The Y chromosome spreads, and more and more individuals will come to carry it; the increasing preponderance of Y over X gametes produced by males carrying the DEG will lead to a population-wide biased sex ratio. This may be good in itself, for example in mosquitoes where only females transmit human diseases, but depending on the extent of the bias and the details of the species’ ecology it may lead to population suppression or even elimination [30, 53]. A number of examples of ‘driving Y chromosomes’ leading to male-biased sex ratios have been observed in nature, though the mechanisms involved are not known [13]. Proof of principle of artificial DEG Y-drive has been shown in the laboratory with mosquitoes [54, 55].

The reason a driving Y spreads can be understood by comparing the number of offspring to which an arbitrarily chosen wild-type and DEG-carrying Y chromosome can expect to be transmitted [20]. Begin by assuming the mating competitiveness and capacity of males carrying the two types of Y chromosome are the same. The wild-type Y will be present in half the offspring that its bearer sires. Because we are assuming equal competitiveness, a male carrying the modified Y will father the same number of offspring (the effect of relaxing this assumption is explored in Additional file 1: Note 17) but the Y:X ratio of its gametes will be 1:1 − *e* where *e* is the probability of successful cleavage (and hence the fraction of X chromosomes destroyed). The modified Y can thus expect to be present in 1/(2 – e) of the offspring, which is always greater than ½. In this simple case, a driving Y chromosome invariably spreads all the way, it can be shown, to fixation.

**Fig. 5.**
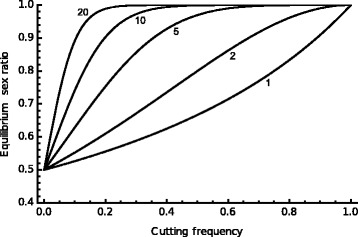
The equilibrium sex ratio resulting from the spread of a DEG on the Y chromosome that cuts the X chromosome as a function of the number of target sites and the probability of cutting [20]

When the cutting frequency is less than one, a DEG that causes multiple breaks increases the sex ratio bias (Fig. 5). More than one break could be engineered by placing several independent DEGs on the Y chromosome or by choosing a DEG that recognised multiple sites on the X chromosome. The latter strategy has been investigated in malaria-vector mosquitoes where the X chromosome carries tandem arrays of rRNA genes with target sites recognised by known homing endonucleases [56]. A single cut in the X chromosome may be repaired by neighbour end joining, which might destroy the recognition site. Multiple cuts are far less likely to be repaired, a further reason to pursue this strategy. Additional file 1: Note 18 discusses in a little more detail resistance to Y drive and how it may be overcome, and Additional file 1: Note 19 and Additional file 3 explore how DEGs that spread by homing and by Y drive could potentially be combined.

## Spatial processes

One of the attractions of gene drive for pest and vector control is that they spread across the landscape: unlike other control methods, such as insecticides or sterile-insect releases, they do not need to be applied everywhere. Modelling is important to give insights into the speed of spread and for the ability of the DEG to spread in spatially heterogeneous regions.

A critical determinant of spatial spread is the extent to which individual insects move in their lifetime. The simplest way to conceptualise this is that an insect that emerges as an adult in one place will move during its lifespan to different places in the landscape with a probability that can be described by a normal distribution centred at its birthplace. The variance of this distribution is proportional to what is called the diffusion coefficient, by analogy to diffusion in physical processes that can be described in the same way [57]. Implicit in this description is the assumption that there is no directional movement or type of behaviour, such as rare long-distance jumps, that would give rise to non-normal distributions. Despite its biological simplicity, it is a good place to start to understand spatial spread.

**Fig. 6.**
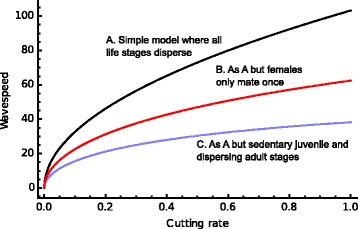
The rate of spread of a DEG through a landscape as a function of homing rate for different assumptions about insect biology (here motivated by mosquitoes) [59]. The wave speed is measured in km year ^−1^, the diffusion parameter is 10 km^−2^ year^−1^, and the adult and juvenile lifespans are 10 and 20 days, respectively. Spread is slower when only some stages disperse and when females mate only once and are thus less efficient at transporting the DEG in sperm

Fisher [58] showed that a beneficial gene will spread through a one-dimensional homogeneous landscape as a travelling wave with speed $2\sqrt{Ds}$ where *D* is the diffusion coefficient and *s* the positive selection coefficient. Spread is faster when organisms disperse more and the gene has a greater advantage. We would expect this insight to apply to DEGs spreading through the landscape and this has been proved rigorously by Beaghton et al. [59] for a driving Y DEG where in the simplest model the rate of spread is $2\sqrt{De}$, where *D* is the diffusion coefficient and *e* is the rate at which the X chromosome is successfully cut by the DEG (as defined in the section on Y Drive). The speed of the travelling wave is not affected by any population suppression caused by the male-biased sex ratio, though this does affect the shape of the travelling wave front: in terms of absolute numbers the driving Y chromosome increases in density before declining to the equilibrium density specified by the non-spatial model. This general result holds when more insect biology is included, but the effect of non-mobile juvenile stages and carriage of the driving Y chromosomes in the stored sperm of mated individuals needs to be taken into account (Fig. 6).

A simple driving DEG that spreads through homing is also likely to spread through a homogeneous landscape at a speed proportional to the square root of the product of average insect movement and the DEG’s net advantage, though this has not been formally demonstrated. As described in Additional file 1: Note 5, some types of DEG are only spread when their frequency exceeds a threshold and here the pattern of spatial spread is through a ‘Bartonian’ rather than a ‘Fisherian’ wave [60] (Additional file 1: Note 20).

The assumption of homogenous populations is clearly unrealistic and results from spatial population ecology will be helpful in analysing these more realistic situations (Additional file 1: Note 21). North et al. [53], for example, developed a stochastic individual-based model of DEG spread (either homing or Y drive) motivated by the biology of mosquitoes. Adults move through the landscape searching for larval oviposition sites or humans on which to feed. When these resources are relatively abundant and uniformly distributed the dynamics of the system can be approximated by non-spatial or spatially homogenous models. When resources are scarcer, an introduced HEG can eliminate a local population and then itself go extinct while wild-type refugia remain. The chance of this happening increases as adult feeding sites become scarce and as the covariance between adult feeding and breeding sites increases: both effects increase the autonomy of the local populations. After the DEG goes extinct the wild-type populations may then recolonize the landscape. The risk of wild-type refuges remaining can be substantially reduced by making multiple releases of DEG-carrying mosquitoes across the environment.

## The future of gene-drive technology

Modelling of gene drive is still relatively in its infancy. Beginning with stylised population genetic and then population dynamic models, theoretical work has expanded to begin to include spatial processes and the ecology of particular species at specific locations. Models have also been developed to explore new tactical possibilities presented by advances in molecular biology, in particular the discovery of the CRISP-Cas9 system.

We believe it highly likely within the next decade that a driving element based on a DEG will be available for deployment to transform or suppress the population of an injurious arthropod, most likely a mosquito vector of an important human disease. Whether deployment actually occurs will depend on whether regulators are satisfied the technology is safe to humans and poses few environmental risks. Modelling will play an important role in submissions to regulators as the genetic and population dynamic consequences of DEG release can be explored in no other way. Deployment will also require a ‘licence to operate’ from civil society, and for citizens to engage profitably in discussions it is critical for modelling outputs to made available in the most accessible forms. This review concentrates on the population biology of gene drive but the same endonucleases that can be used in gene drive may also be used in contexts where spread does not occur or is limited, for example in genetic versions of the sterile insect technique (Additional file 1: Note 22). It is likely that these will be the first modified endonucleases permitted to be released.

**Fig. 7.**
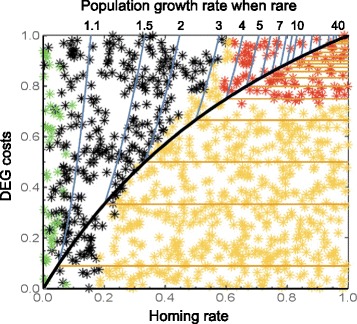
Results of a detail-rich simulation of the spread of a costly DEG through a population of the mosquito *Anopheles gambiae* (*sensu stricto*) based on population parameters and weather at a site in Tanzania (from [61]). The colours of the *stars* are the results of simulations for the particular cost and homing frequency combinations: *green*, DEG fails to establish; *yellow*, DEG goes to fixation and population persists; *black*, equilibrium with both DEG and wild-type allele; *red*, population elimination. Also shown is the degree to which simple models explain these results. Genetic models predict the DEG always invades and is fixed for parameter values below the *thick black line* [20]. Because of stochastic effects, when homing is very weak the DEG is often lost by chance and fails to establish (*green stars* at *left*). Also, when homing is very weak, it may take a very long time for fixation to occur, which explains the *black stars* to *lower left*. Population models predict elimination in a *top right sector* whose size depends on the growth rate of the population when rare [30]. This was not estimated explicitly from the data but a value of around 4 (geometric mean over time) best fits the simulation. When the population is small and near elimination the DEG may get fixed through random effects, which explains the *yellow stars* above the *line* near the region of elimination in the *top right*. Overall there is a pleasing concordance between the results of the simple and complex modelling exercises

Looking ahead we see two major developments in modelling DEG dynamics. Most of the work discussed in this review has involved fairly abstract and mathematically tractable models. Increasingly, as particular vectors of disease or pests are targeted for intervention, much more complex simulation models will be developed, for example incorporating substantial details of the vector-disease system concerned in specific spatial settings. A recent model of African malaria transmitted by *Anopheles gambiae* by Eckhoff et al. [61] is perhaps the first of this type and, encouragingly, gives results largely in agreement with those of much simpler analytical models (Fig. 7; Additional file 1: Note 23). We are also likely to see more studies of particular deployment strategies (for example the timing, location and size of releases; Additional file 1: Note 24).

The field has changed enormously in the mere five years since CRISPR-Cas9 burst onto the scene [16] and it would be rash to predict how gene drive technology may be affected by new molecular advances in the coming years. Conceptually, CRISPR-Cas9 DEGs function largely in the same way as the first generation of homing endonucleases and much of the theory developed for this first generation of DEGs is equally applicable. But the ease of manipulation of CRISPR systems is transformational and has brought new laboratories and new funds into the field, as well as new suggestions about how DEGs may be deployed. It has also led to greatly increased press and public attention, and the field, rightly, will be expected to be transparent and responsible [62]. This is a uniquely exciting time to work on the genetic control of pests and vectors.

## Additional files

Additional file 1: Includes 24 notes that provide further explanation or details of material presented in the main text. (DOCX 103 kb)

Additional file 2: A compiled computer programme written in *Mathematica,* that allows further exploration of the CATCHA mechanism (see Additional file 1: Note 16). (CDF 44 kb)

Additional file 3: A compiled computer programme that allow classic homing gene drive and driving Y chromosomes to be explored separately and together (see Additional file 1: Note 19). (CDF 144 kb)

## References

[1] Sinkins SP, Gould F (2006). Gene drive systems for insect disease vectors. Nat Rev Genet.

[2] Burt A (2014). Heritable strategies for controlling insect vectors of disease. Philos Trans R Soc B Biol Sci.

[3] Esvelt KM, Smidler AL, Catteruccia F, Church GM. Concerning RNA-guided gene drives for the alteration of wild populations. Elife. 2014;3:e03401. 10.7554/eLife.03401 PMC4117217 25035423

[4] Champer J, Buchman A, Akbari OS (2016). Cheating evolution: engineering gene drives to manipulate the fate of wild populations. Nat Rev Genet.

[5] Gantz VM, Bier E (2016). The dawn of active genetics. Bioessays.

[6] Alphey L (2016). Can CRISPR-Cas9 gene drives curb malaria?. Nat Biotechnol.

[7] Adelman ZN, Tu ZJ (2016). Control of mosquito-borne infectious diseases: sex and gene drive. Trends Parasitol.

[8] Leftwich PT, Bolton M, Chapman T (2016). Evolutionary biology and genetic techniques for insect control. Evol Appl.

[9] Craig GB, Hickey WA, van de Hey RC (1960). An inherited male-producing factor in *Aedes aegypti*. Science.

[10] Hickey WA, Craig GB (1966). Genetic distortion of sex ratio in a mosquito, *Aedes aegypti*. Genetics.

[11] Curtis CF (1968). Possible use of translocations to fix desirable genes in insect pest populations. Nature.

[12] Hamilton WD (1967). Extraordinary sex ratios. Science.

[13] Burt A, Trivers R (2006). Genes in conflict.

[14] Stoddard BL (2005). Homing endonuclease structure and function. Q Rev Biophys.

[15] Burt A (2003). Site-specific selfish genes as tools for the control and genetic engineering of natural populations. Proc Biol Sci.

[16] Jinek M, Chylinski K, Fonfara I, Hauer M, Doudna JA, Charpentier E (2012). A programmable dual-RNA-guided DNA endonuclease in adaptive bacterial immunity. Science.

[17] Doudna JA, Charpentier E (2014). The new frontier of genome engineering with CRISPR-Cas9. Science.

[18] Simoni A, Siniscalchi C, Chan YS, Huen DS, Russell S, Windbichler N (2014). Development of synthetic selfish elements based on modular nucleases in Drosophila melanogaster. Nucleic Acids Res.

[19] Fauci AS, Morens DM (2016). Zika Virus in the Americas - yet another Arbovirus threat. New Engl J Med.

[20] Deredec A, Burt A, Godfray HCJ (2008). The population genetics of using homing endonuclease genes in vector and pest management. Genetics.

[21] Goddard MR, Burt A (1999). Recurrent invasion and extinction of a selfish gene. Proc Natl Acad Sci U S A.

[22] Windbichler N, Menichelli M, Papathanos PA, Thyme SB, Li H, Ulge UY (2011). A synthetic homing endonuclease-based gene drive system in the human malaria mosquito. Nature.

[23] Hammond A, Galizi R, Kyrou K, Simoni A, Siniscalchi C, Katsanos D (2016). A CRISPR-Cas9 gene drive system-targeting female reproduction in the malaria mosquito vector Anopheles gambiae. Nat Biotechnol.

[24] Gantz VM, Jasinskiene N, Tatarenkova O, Fazekas A, Macias VM, Bier E (2015). Highly efficient Cas9-mediated gene drive for population modification of the malaria vector mosquito *Anopheles stephensi*. Proc Natl Acad Sci U S A.

[25] Burt A, Koufopanou V (2004). Homing endonuclease genes: the rise and fall and rise again of a selfish element. Curr Opin Genet Dev.

[26] Koufopanou V, Goddard MR, Burt A (2002). Adaptation for horizontal transfer in a homing endonuclease. Mol Biol Evol.

[27] Unckless RL, Messer PW, Connallon T, Clark AG (2015). Modeling the manipulation of natural populations by the mutagenic chain reaction. Genetics.

[28] Unckless RL, Clark AG, Messer PW (2017). Evolution of resistance against CRISPR/Cas9 gene drive. Genetics.

[29] Noble C, Olejarz J, Esvelt K, Church G, Nowak M. Evolutionary dynamics of CRISPR gene drives. Sci. Advances. 2017;3(4):e1601964. 10.1126/sciadv.1601964 PMC5381957 28435878

[30] Deredec A, Godfray HCJ, Burt A (2011). Requirements for effective malaria control with homing endonuclease genes. Proc Natl Acad Sci U S A.

[31] Marshall JM, Buchman A, Sánchez HM, Akbari OS. Overcoming evolved resistance to population-suppressing homing-based gene drives. Sci Rep-Uk. 2016;in press. 10.1038/s41598-017-02744-7 PMC5476637 28630470

[32] Molineaux L, Gramiccia G (1980). The Garki Project.

[33] Tuljapurkar S (1990). Population dynamics in variable environments.

[34] Stephens PA, Sutherland WJ, Freckleton RP (1999). What is the Allee effect?. Oikos.

[35] Gimnig JE, Ombok M, Otieno S, Kaufman MG, Vulule JM, Walker ED (2002). Density-dependent development of *Anopheles gambiae* (Diptera: Culicidae) larvae in artificial habitats. J Med Entomol.

[36] White MT, Griffin JT, Churcher TS, Ferguson NM, Basanez MG, Ghani AC (2011). Modelling the impact of vector control interventions on *Anopheles gambiae* population dynamics. Parasites Vectors.

[37] Muriu SM, Coulson T, Mbogo CM, Godfray HCJ (2013). Larval density dependence in *Anopheles gambiae* s.s., the major African vector of malaria. J Anim Ecol.

[38] May RM (1974). Stability and complexity in model ecosystems.

[39] Alphey N, Bonsall MB (2014). Interplay of population genetics and dynamics in the genetic control of mosquitoes. J R Soc Interface.

[40] Anderson RM, May RM (1991). Infectious diseases of humans.

[41] Macdonald G (1957). The epidemiology and control of malaria.

[42] Smith DL, McKenzie FE (2004). Statics and dynamics of malaria infection in *Anopheles* mosquitoes. Malaria J.

[43] Cook PE, McMeniman CJ, O'Neill SL (2008). Modifying insect population age structure to control vector-borne disease. Adv Exp Med Biol.

[44] Hancock PA, Thomas MB, Godfray HCJ (2009). An age-structured model to evaluate the potential of novel malaria-control interventions: a case study of fungal biopesticide sprays. Proc Biol Sci.

[45] Koella JC, Lynch PA, Thomas MB, Read AF (2009). Towards evolution-proof malaria control with insecticides. Evol Appl.

[46] Lyimo EO, Koella JC (1992). Relationship between body size of adult *Anopheles gambiae* s.1. and infection with the malaria parasite *Plasmodium falciparum*. Parasitology.

[47] Weterings E, Chen DJ (2008). The endless tale of non-homologous end-joining. Cell Res.

[48] Yajima H, Fujisawa H, Nakajima NI, Hirakawa H, Jeggo PA, Okayasu R (2013). The complexity of DNA double strand breaks is a critical factor enhancing end-resection. DNA Repair.

[49] Neafsey DE, Waterhouse RM, Abai MR, Aganezov SS, Alekseyev MA, Allen JE (2015). Highly evolvable malaria vectors: the genomes of 16 Anopheles mosquitoes. Science.

[50] Beaghton A, Hammond A, Nolan T, Crisanti A, Godfray HCJ, Burt A (2017). Requirements for driving anti-pathogen effector genes into populations of disease vectors by homing. Genetics.

[51] Huang YX, Magori K, Lloyd AL, Gould F (2007). Introducing desirable transgenes into insect populations using Y-linked meiotic drive--a theoretical assessment. Evolution.

[52] Wu B, Luo LQ, Gao XJJ (2016). Cas9-triggered chain ablation of cas9 as a gene drive brake. Nat Biotechnol.

[53] North A, Burt A, Godfray HCJ (2013). Modelling the spatial spread of a homing endonuclease gene in a mosquito population. J Appl Ecol.

[54] Galizi R, Doyle LA, Menichelli M, Bernardini F, Deredec A, Burt A (2014). A synthetic sex ratio distortion system for the control of the human malaria mosquito. Nat Commun.

[55] Galizi R, Hammond A, Kyrou K, Taxiarchi C, Bernardini F, O'Loughlin SM, et al. A CRISPR-Cas9 sex-ratio distortion system for genetic control. Sci Rep. 2016;6:31139. 10.1038/srep31139 PMC4971495 27484623

[56] Windbichler N, Papathanos PA, Catteruccia F, Ranson H, Burt A, Crisanti A (2007). Homing endonuclease mediated gene targeting in *Anopheles gambiae* cells and embryos. Nucleic Acids Res.

[57] Ovaskainen O, de Knegt HJ, del Mar Delgado M (2016). Quantitative ecology and evolutionary biology.

[58] Fisher RA (1937). The wave of advance of advantageous genes. Ann Hum Genet.

[59] Beaghton A, Beaghton PJ, Burt A (2016). Gene drive through a landscape: Reaction-diffusion models of population suppression and elimination by a sex ratio distorter. Theor Popul Biol.

[60] Barton NH (1979). The dynamics of hybrid zones. Heredity.

[61] Eckhoff PA, Wenger EA, Godfray HCJ, Burt A (2017). Impact of mosquito gene drive on malaria elimination in a computational model with explicit spatial and temporal dynamics. Proc Natl Acad Sci U S A.

[62] Esvelt K (2016). Gene editing can drive science to openness. Nature.

