# Supplementary material for: How driving endonuclease genes can be used to combat pests and disease vectors
Source: BMC Biol. 2017 Sep 11;15:81. doi: 10.1186/s12915-017-0420-4 (PMC5594614; doi:10.1186/s12915-017-0420-4)
Supplement: Supplementary file 1 — Includes 24 notes that provide further explanation or details of material presented in the main text. (DOCX 103 kb) [file 12915_2017_420_MOESM1_ESM.docx]

# How driving endonuclease genes can be used to combat pests and disease vectors

H. Charles J. Godfray^1^, Ace North^1^ and Austin Burt^2^

^1^ Department of Zoology, University of Oxford, South Parks Road, Oxford OX1 3PS, United Kingdom

^2^ Department of Life Sciences, Imperial College London, Silwood Park, Ascot, Berkshire SL5 7PY, United Kingdom

# Additional File 1

## Additional file 1: Note 1. Is CRISPR different?

Much of the recent upsurge in interest in DEGs is due to the discovery and rapid adaptation of CRISPR-Cas9 and related endonuclease systems, which in many ways are much easier to manipulate than homing endonuclease genes (HEGs) and other earlier DEGs [1–3]. But do they differ conceptually from these earlier constructs? For the most straightforward applications of CRISPR to gene drive, clearly not; the theory developed for HEGs can be applied without modification. Similarly, they are as prone to the emergence of resistance alleles, perhaps more so because of the complexity of the molecular machinery that has to be copied from one chromosome to the other during homing [4]. Again, the basic dynamics of resistance to CRISPR can be analysed in the same way as earlier systems [5, 6].

CRISPR systems direct the Cas9 endonuclease to cut the chromosome at sequences determined by guide RNAs. An advantage of CRISPR over HEGs and other methods is that multiple guide RNAs can in principle be combined together (multiplexed) to cut the same gene in multiple places, so reducing the likelihood of resistance occurring. Esvelt et al. [7], Unckless et al. [8] and Marshall et al. [4] (see also the section on Resistance and recall) explore some of the molecular considerations involved in designing multiplexed gene drives and Esvelt et al. [7] review other ways in which the molecular biology of CRISPR-Cas9 can be adapted to improve drive prospects. The latter involves designing drive constructs that interfere with non-homologous end joining (EJ) to increase the probability of homology-directed repair [9], and reducing the risk of partial homing of the drive construct by reducing homology away from the target site. The impact of these critically important molecular biological issues can be analysed by seeing how they affect the parameters of the standard models of gene drive.

## Additional File 1: Note 2. Sensitising drive

Many of the potential target species for gene drive are currently controlled by insecticides, acaricides or other pest-control chemicals. The evolution of resistance to these compounds is a major reason for studying gene drives. Esvelt et al. [7] have considered whether gene drive might be used to reverse the evolution of resistance or to introduce a construct that sensitises the species to an existing or novel compound that could be delivered using standard chemical application methods. These possibilities have not been formally modelled but the genetics would be standard and the population dynamics determined by the extent and frequency of spraying. The possible logic for pursuing this strategy is that population suppression would be limited to where the spraying occurs, but a barrier is that it combines the inefficiency of traditional control methods with the regulatory challenges of novel genetic methods.

## Additional file 1: Note 3. Net costs and benefits

The arbitrarily chosen individual wild-type allele will be present in a wild-type homozygote with probability (1 – *q*) and be transmitted to half the offspring. It will be in a heterozygote with probability *q* and present in ½(1 – *e*) of the offspring. An arbitrarily chosen DEG will be in a heterozygote with probability (1 – *q*) and present in ½(1 + *e*) of the offspring, and in the homozygote with probability *q* and present in half of the diminished (1 – *s*) offspring (where *s* is fitness costs). Equating wild-type and DEG offspring production we obtain:

½ (1 – *q*) + ½ *q* (1 – *e*) = ½ (1 – *q*) (1 + *e*) + ½ *q* (1 – *s*).

Multiplying by 2 and subtracting *q* + (1 – *q*) = 1 from both sides gives the exactly equivalent condition which can be interpreted in terms of the effects (costs and benefits) of the presence of the DEG on the two alleles:

– *q* *e* = (1 – *q*) *e* – *q s*.

## Additional file 1: Note 4. Sex-specific expression

DEGs can be designed to target sex-specific fertility or viability genes. To understand the spread of such a DEG it is necessary to keep track of gene frequencies in each generation in the two sexes, which makes the mathematics harder to analyse and more difficult to appreciate intuitively.

The speed of spread of a fully recessive DEG, and its frequency at equilibrium if it does not go to fixation, is a balance between the efficiency with which it homes and losses due to homozygote costs. Restricting those costs to one sex is beneficial to the DEG and the speed of spread, likelihood of fixation, and equilibrium gene frequency all increase. Where a costly DEG is being introduced to suppress population density it clearly makes most sense to target female viability or fertility because population density is much less affected by male mortality or infertility, except perhaps in species where mating with an infertile male is equivalent to being infertile for a female. The frequency of homozygotes is *q_m_ q_f_*, the product of the sex-specific gene frequencies (*q_m_* and *q_f_*), and hence the load is 1 – *q_m_ q_f_* *s*, which can be shown to be always greater than 1 – *q*^2^*s*, the sex-symmetric case (recall we assume that the gene is expressed before homing so only homozygotes suffer fitness costs). The general result that female-specific costs increase genetic loads applies more broadly to cases where heterozygotes suffer fitness costs. If the aim is population replacement and finding a DEG with minimum costs is the goal, then other things being equal a construct that had a negative effect on the fitness of one rather than both sexes would be preferable.

Other sex-specific effects are possible, for example sex-specific homing rates. However, DEG performance is roughly determined by the mean homing frequency in the two sexes. More complicated combinations of sex-specific effects are possible, with some leading to much reduced spread. For example, if homing can only be achieved in one sex then targeting fertility genes in that sex is a much better strategy than imposing a cost on the other sex. Were it possible to find a gene that renders both males and females infertile so that the only viable mating combinations are those where neither parent is homozygote DEG, a very high genetic load would result.

For further details on sex-specific effects see Deredec et al. (5).

## Additional file 1: Note 5. Heterozygote costs

In this note we explore the dynamics of DEGs that have effects on fitness in the heterozygote (continuing to assume expression before homing in heterozygores), concluding with a summary of how they may influence the design of population suppression and replacement strategies.

To explore the dynamics of a DEG that affects fitness in the heterozygote it is helpful to recognise two threshold values in homing frequency. The first is the threshold for spread to occur (call it *e*_1_) and the second is the threshold above which the DEG always goes to fixation (call it *e*_2_). Recall that in the case of no costs to the heterozygote, we showed above that the DEG always spread so that the threshold for spread was zero while the threshold for fixation equalled the selection acting against the homozygote DEG (*e*_2_ = *s*).

The spread of a DEG occurs when homing is frequent enough that it more than compensates for the reduced number of offspring produced by the heterozygote. For a fixed cost of the homozygote DEG we can trace what happens as the cost to the heterozygote increases from zero to being equal to the homozygote DEG (or to put it another way as the functional gene targeted by the HEG moves from fully dominant to fully recessive).

As before, assume the fitness of the DEG homozygote is 1 – *s* but now assume the fitness of the heterozygotes is 1 – *h s* where *h* varies from 0 (DEG fully recessive) to 1 (DEG fully dominant). An arbitrarily chosen rare DEG can then expect to produce ½ (1 + *e*) (1 − *h s*) copies of itself which must be greater than ½ (the number of copies an arbitrarily chosen rare wild-type allele will produce) for spread to occur. This expression shows the tension between higher *e*, which makes spread more likely, and higher *h s*, which has the opposite effect. Equating and rearranging we get the threshold for spread *e*_1_ = *h s*/(1 – *h s*), which is zero when *h* = 0.

We can again look for where the net costs and benefits of the presence of the DEG are the same for both alleles to identify the equilibrium DEG frequency (*q*). A wild-type allele will find itself in a heterozygote with probability *q* where its relative fitness is (1 – *h s*)(1 – *e*); subtracting 1 from this gives the net cost or benefit. The equivalent expression for the DEG in a heterozygote (where it will be with probability (1 – *q*) is (1 – *h s*)(1 + *e*) – 1 and for a DEG in a homozygote (probability *q*) it is – *s* (as before). Putting these together we get,

– *q* [(1 – *h s*)(1 – *e*) – 1] = (1 – *q*) [(1 – *h s*)(1 + *e*) – 1] – *q* s.

Solving for *q* we obtain the equilibrium

*q* = (*e* – (1 + *e*) *h s*)/((1 – 2*h*) *s*).

This exactly equals one when *e*_2_ = *s* (1 – *h*)/(1 – *h s*), which is the second threshold (from which we can see that *e*_2_ = *s* when *h* = 0 as we derived before).

Note that fixation occurs for weaker homing as heterozygote costs mount. This occurs because when the DEG becomes common and more and more of the wild-type alleles which remain are found in heterozygotes, they suffer the double disbenefit of lower fitness and the risk of conversion to a DEG.

A new phenomenon emerges as heterozygote fitness drops. For low heterozygote costs the DEG fails to spread for homing rates between 0 and *e*_1_, a stable polymorphism results in the interval between *e*_1_ and *e*_2_, and the DEG is fixed between *e*_2_ and 1. As heterozygote costs increase the middle interval becomes squeezed until the heterozygote fitness is the average of the two homozygotes when *e*_1_ = *e*_2_. Now the DEG either fails to invade or becomes fixed depending on whether the homing rate is above or below this single threshold. What happens if heterozygote costs increase further, approaching those of the DEG homozygote? The threshold *e*_1_ continues to increase with fixation always occurring when it is exceeded, while *e*_2_ drops, the DEG failing to establish when homing rates are lower. Between these two thresholds (with now *e*_2_ < *e*_1_) the DEG *either* fails to spread *or* becomes fixed, but with the outcome depending on the initial frequency of the DEG. Recall that the DEG spreads when an arbitrarily chosen DEG allele produces more copies of itself than an arbitrarily chosen wild-type allele. When heterozygote costs are high, spread may only occur when the DEG is sufficiently common that a large fraction of the wild-type alleles are in heterozygotes and share some of the costs invariably experienced by the DEG.

How do heterozygote costs affect the design of gene-drive strategies? First, moderate costs should be seen as no impediment when drive is relatively strong. Second, when population replacement is the aim and a choice is available, minimising fitness costs (in both the heterozygote and homozygote) will make spread easier. Third, for population suppression, heterozygote costs are likely to make establishment harder, but once established can lead to greater population suppression. Finally, when the target gene is recessive, successful deployment of a DEG may require releases to be sufficiently large that DEG frequencies in the field exceed a threshold. A DEG of this last type is similar to other proposed gene drive mechanisms (such as overdominant chromosomes [10]) that also only spread above a threshold. It has been suggested that such genes may be useful when easy containment is an objective and limitations on spread an advantage (see also Additional file 1: Note 22)

## Additional file 1: Note 6. Timing of expression

So far we have assumed that the DEG converts a heterozygote to homozygote after any gene at the target site is expressed. This means that any costs of being a homozygote (*s*) are not visited on the individual in which homing occurs. What happens when homing occurs first? Again consider a rare DEG allele, which will almost certainly be in a heterozygote: will it be transmitted to more than half its bearer offspring? In a fraction 1 – *e* of cases homing will not occur and the particular gene, like its wild-type alternative, will be transmitted to half the offspring. In a fraction *e* of cases homing occurs with two consequences: fitness is reduced and the number of offspring is lower by 1 – *s*, but the DEG allele is transmitted to all offspring. Bringing together these two alternatives, spread will occur if ½ (1 – *e*) + *e* (1 – *s*) > ½, which reduces to the condition *s* < ½. Spread from rare cannot occur when the DEG reduces fitness by more than 50 %.

Exactly the same analysis of the full dynamics including heterozygote fitness effects can be carried out as before. When fitness costs are very low there is little difference in the dynamics, and such a DEG would be suitable for population replacement. When costs are higher, spread is harder to achieve compared to when the DEG is active after gene expression, and for the same parameters the load exerted in the population is lower.

The timing of expression is very important for population suppression strategies, where the aim is to impose a high load, and the genes that best do this cannot spread if homing occurs before expression. It is much less significant in population replacement strategies where the DEG is designed to have low costs. This difference in emphasis explains why Deredec et al. [5, 6], motivated by population suppression, concentrated on the case of homing after expression while several more recent groups [8, 11, 12], thinking of population replacement, build models where homing precedes expression.

## Additional file 1: Note 7. Speed of spread

How many generations does it take for a DEG to spread? The speed of spread is initially little influenced by costs for the case of a completely recessive DEG as most copies of the gene are in heterozygotes. We find that a good approximation for the number of generations it takes for the DEG to reach a frequency of 0.5 is *M*/Log_10_(1 + *e*), where *M* is the number of orders of magnitude the gene has to increase from its starting frequency (so if after release the frequency of the DEG is 0.5 × 10^−6^ then *M* = 6). The homing frequency, *e*, must lie between 0 and 1. For low *e* the quantity Log_10_(1 + *e*) is very small and hence the number of generations is very large. But when *e* is at its maximum value the number of generations is ~3.3*M* (and for *e* = 0.9 it would be ~3.6*M*). DEGs of this type with high homing frequencies can spread from very low densities in around 20 to 30 generations. Having achieved a frequency of 0.5, DEGs with no fitness costs approach fixation rapidly while those with substantial fitness costs take somewhat longer.

## Additional file 1: Note 8. Stochasticity

Of course population growth rates do not stay constant over time but vary both haphazardly and seasonally. Population genetic study of the spread of beneficial genes in temporally variable environments would suggest that the condition for elimination is likely to be of the form $L>1-1/\bar{R}_{m}$, where the bar denotes the *geometric* (not the arithmetic) mean value of *R_m_* over time. This is supported by simulation models of the potential deployment of specific DEGs [13], though further insights from more general models would be helpful. A large literature on extinction and stochastic population dynamics, much developed by conservation biologists seeking to avoid this eventuality, is relevant to DEG population suppression strategies [14].

## Additional file 1: Note 9. Allee effects

There are some populations whose reproductive rate does not keep increasing as populations decline and competition for resources abate. A possible reason for this is that when individuals are sparsely distributed across the environment it is hard to find mates. In these species there may be a population density threshold below which the species is unable to recover and elimination is inevitable; ecologists call this an Allee effect. Most examples of Allee effects come from vertebrates [15], though some bark beetles can only overcome host tree defences when sufficient insects attack [16]. The presence of an Allee effect is likely to increase the probability of population elimination occurring.

## Additional file 1: Note 10. Complex dynamics

In reality the factors affecting population size are much more complex than this very simple model. Density-dependent mortality may occur in several parts of the life cycle, and may vary greatly in magnitude, both seasonally and between years. Some species’ population dynamics may best be described as a random walk around a gently rising trend, buffeted by mortality that acts irrespective of density, that only occasionally hits a population density ceiling where density-dependent processes come into effect. Density-dependent and density-independent mortality will also vary over space. Generalisations are difficult and were deployment of a DEG to be considered, tactical models targeted at individual species’ ecology will be required. These are beginning to be developed for mosquito vectors of disease [13].

## Additional file 1: Note 11. Species interactions

Any species targeted by a DEG intervention will be part of a food web, predated and parasitised by other organisms, and potentially competing with other species for resources. Might the elimination or suppression of a target species cause ecological perturbations, at the worst leading to unexpected negative effects? This again is not a specific issue for DEGs but for any intervention seeking to drive down the number of a pest or vector. In some ways the question is easier to answer for a DEG whose action is limited to a single species (as opposed, for example, to a broad spectrum insecticide), though alternative interventions seldom result in elimination.

These questions can be explored using some of the many population models for species interactions developed by theoretical ecologists. The issue is not so much the analysis but the lack of information available to guide their development. An important question that has been raised several times is whether targeting a human disease vector might lead to an empty ecological niche that is then colonised by another species that is a worse vector [17]. This can only be answered on a case-by-case basis though for *Anopheles gambiae*, the major vector of malaria in Africa, consideration of what is known about its ecology and the vectorial capacity of the species with which it competes suggests it is unlikely.

## Additional file 1: Note 12. R_0_

The basic epidemiological number, *R*_0_, is the number of secondary infections that occur due to a single infection that arises in a susceptible population. If an intervention reduces *R*_0_ to below 1 the disease will fail to establish and die out. There is a vast literature on *R*_0_ for different vector-borne diseases (e.g. [18]) that will be relevant for using DEGs to target vectors. Here we illustrate this using the standard formulation for *R*_0_ and malaria.

Assume a human has been infected by malaria and can now transmit the disease to a mosquito that feeds on him or her. To calculate the probability that this occurs we need to know (i) how long the person remains infectious; (ii) the likelihood of being fed on by a mosquito; and (iii) the probability of a susceptible human becoming infected upon being bitten by an infectious mosquito. The first quantity is typically rendered as 1/*r* where *r* is the probability per day of ceasing to be infectious (due to clearance or death). The second is *a m* where *a* is the rate at which female mosquitoes bite humans per day and *m* is the density of mosquitoes divided by the density of humans (in effect assuming the mosquito population exactly divides up the human population). Finally, the third quantity is taken to be a constant, *b*. Once in the mosquito the malaria pathogen has to migrate from the gut to the salivary gland and mature, a process that typically takes about 10 days (the extrinsic incubation period). If the mosquito suffers daily mortality with probability *g* (assumed as a first approximation not to vary with age) then for there to be any chance of disease transmission the mosquito must survive for *n* days, which occurs with probability (1 − *g*)*^n^*. After this period the mosquito can transmit the disease and can expect to live for a further 1/*g* days (lifespan is the reciprocal of the mortality rate). Over this period it will feed on a human with probability *a* per day (and as the disease has only just been introduced into the population we can assume the person is not previously infected) and when it feeds we let the probability this results in the *Plasmodium* being successfully transmitted to the human be *c*. Linking together this chain of possibilities we obtain the expression for *R*_0_:

$R_{0}=m\left[ \frac{a^{2}bc{(1-g)}^{n}}{rg} \right]$.

This formula is valuable as it shows how different DEG strategies may work to eliminate or reduce the effects of a disease. Population suppression strategies aim to reduce mosquito densities *m*: if the vector is eliminated then *m* = 0 and the disease dies out but this also occurs if *m* is reduced sufficiently that *R*_0_ falls below one. The equation shows that whether the latter occurs depends in quite a complicated way on the detailed biology of the system. Population replacement strategies seek to spread a construct that blocks or reduces transmission, either from human to mosquito (reducing *b*) or in the opposite direction (reducing *c*). The expression for *R*_0_ indicates the functional equivalence of both strategies: *ceteris paribus* a 50 % reduction in mosquito numbers (*m*) would have the same effect as a 50% reduction in transmission (*b* or *c*). A DEG that affected the risk of adult mortality (*g*) would have a much more complicated effect. Higher mortality would influence the number of mosquitoes available to bite humans (*m*) but also reduce transmission as fewer mosquitoes would survive the intrinsic incubation period (*n*^1-^*^g^* is lower) and those that did would live to transmit the disease for a shorter period of time (1/*g* is also lower).

Consideration of *R*_0_ can also be useful for exploring how different vector control measures can combine to control disease. For further details about how analyses of *R*_0_ can inform intervention strategies see Brady et al. [19, 20].

## Additional file 1: Note 13. When resistance has fitness costs

The dynamics of resistance mutations are more complicated if they do not fully restore wild-type fitness. One possibility is that EJ destroys the recognition site but does not restore fitness—the result is a non-functional allele but one that does not drive. In the simplest case of a lethal homozygote DEG (where, as described in the main text, the load is *e*^2^ at equilibrium) the load is reduced by a factor (1 – *m*)^2^, where *m* is the fraction of homing events that give rise to mutant alleles. This type of misrepair might also stop a cost-free DEG used for population replacement going to fixation. However, in both cases the effect is small unless misrepair is very common. A full analysis of the dynamics of escape mutants that have fitness intermediate between the wild-type and the DEG, and for different types of heterozygote fitness, has not been performed. Examination of specific simulations suggests that the nearer escape mutations are to wild-type fitness the faster they spread, though it is possible to choose parameter combinations exhibiting complex dynamics such as persistent cycles and dependence on initial conditions [5].

## Additional file 1: Note 14. DEGs that cut the target gene at multiple sites

Marshall et al. [4] determined the minimum rate of mutation to a resistance allele ($\hat{\rho}$) that was compatible with the elimination of populations of different size. They had in mind a mosquito vector of malaria and used a model based on Deredec et al. [6] and assumed an intrinsic rate of population growth of between 2 and 12. Rates of EJ observed in the laboratory for CRISPR based systems ($\rho$ = 10^−2^) [3] (though note these may reduce as the technology matures) were sufficiently high that only temporary dips in the density of moderately sized populations (*N* ≈ 10^4^) would be expected before resistance alleles became established. Elimination would only occur if resistance mutation rates were substantially lower ($\hat{\rho}\approx$10^-4^). Larger populations required ever lower mutation rates, the relationship being linear on a log scale ($\mathrm{Log}\left( \hat{\rho} \right)\propto-\mathrm{Log}(N))$, leading to the conclusion that if resistance alleles are not very costly no single DEG is likely to be able to eliminate a pest or vector population of a size likely to be encountered in the field. However, if the DEG cut the target in multiple places such that all sites must become resistant then elimination becomes feasible. Now it is the product of target site number (*m*) and log mutation rate that determines whether elimination occurs ($m \mathrm{Log}\left( \hat{\rho} \right)\propto-\mathrm{Log}(N))$ and a relative small number of multiple target sites can serve to prevent escape alleles arising before even very large populations are eliminated.

## Additional file 1: Note 15. Resistance under population replacement

Noble et al. [12] explore the rate at which resistance arises and its increasing likelihood as population size gets larger. They derive a valuable framework for analysing CRISPR-based systems with arbitrary numbers of guide RNAs targeted on the same gene. Their analysis focuses on population replacement strategies where any individual carrying a DEG (either one or two copies) suffers a fitness cost (perhaps through non-target effects of the drive mechanism) and where the DEG homes by targeting a gene that, if both copies are disrupted, imposes a further cost on the organism (see also [8, 21]). An interesting twist they add is to model the suggestion [7] that the DEG should be linked to a functional copy of the target gene that is immune to cutting (by altering the DNA sequence using only synonymous sites) and that drives through the population in tandem. This reduces the disadvantage of the homozygous DEG and hence selection for resistance. They compared the length of time a simple DEG remains in a population before resistance arises with the persistence of an enhanced DEG with multiple (5) guide RNAs and with the resistant version of the target gene as its linked cargo. The redesigned construct performs considerably better.

## Additional file 1: Note 16. CATCHA

If a DEG has gone to fixation then the dynamics of the CATCHA allele are precisely the same as the dynamics of a DEG spreading through a wild-type population (see main text and Additional file 1: Note 5). The CATCHA allele is at an advantage in the heterozygotes as it suborns the CRISPR machinery of the alternative allele to copy itself in place of the Cas9 gene. As Wu et al. [22] note, the dynamics may be more complex when the wild-type allele is present and where genotype costs vary and in a separate file (Additional file 2) we provide a compiled *Mathematica* programme to allow these possibilities to be explored.

## Additional file 1: Note 17. Sperm dynamics

The destruction of the X chromosome may mean that the individual male carrying the driving Y produces half the viable sperm of the wild-type (though depending on the details of the X inactivation it is possible that the male redirects resources towards the remaining viable and largely Y-bearing sperm). If the male mates with a single female this may not matter as typically one copulation provides many more sperm than are needed to fertilise all a female’s eggs. But if a female mates with many males then the relative advantage of a rare driving Y not having to compete with ‘sister’ Xs is diluted by the presence of other ejaculates containing full complements of wildtype X and Y gametes. The spread of a driving Y will also be affected by any collateral negative effects on male fitness, perhaps off-target effects of the nuclease that reduce survival or mate competitiveness. Whether these effects will prevent invasion by a driving Y can be determined by asking if a driving Y chromosome when rare will still be transmitted more frequently than an arbitrarily chosen wild-type Y. When invasion does occur, the driving Y will increase in frequency until it becomes fixed, though more slowly than in the unconstrained case [5].

## Additional file 1: Note 18. Multiple Y-drive DEGs and Y-drive resistance

The mathematics of the two strategies of placing several independent DEGs on the Y chromosome and choosing a DEG that recognises multiple sites on the X chromosome is in principle the same, though the precise dynamics may be affected by the details of the molecular biology. Assuming all cuts are made with the same frequency, *e*, then the chromosome survives one cut with probability (1 – *e*) and *k* cuts with probability (1 – *e*)*^k^* and the driving Y spreads if 1/[ 1 + (1 – *e*)*^k^*] is greater than ½. Stacking multiple copies of even relatively inefficient DEGs can, as long as they act independently to ‘shred’ the X chromosome, result in a very efficient drive mechanism.

Successful EJ that does not destroy the DEG recognition site is equivalent to a reduction in cleavage efficiency (*e*). But if the recognition site is destroyed then an escape mutant is generated. Its spread can be understood by comparing its transmission frequency with a wild-type X chromosome. In the absence of any costs it clearly is more likely to be transmitted as it is immune from cutting. Such an escape mutant would increase in frequency until it became fixed and the sex ratio would return to equality. If the recognition site is within a gene required for successful X function then an escape mutant might have reduced fitness relative to the wildtype X. A comparison of transmission rates will again say whether it spreads but its ultimate fate (fixation or polymorphism, equal or biased sex ratios) is more complex and depends on the relative costs of the escape-mutant X (which may vary in males and females) and the driving Y relative to their wild-type counterparts [5].

Multiple cuts are less likely to be mended by EJ and hence this strategy increases resilience to resistance. An escape mutant would need to act systemically, interfering with the DEG or DEGs before they made their cuts. Were such a mechanism to arise on the X chromosome, the conditions for spread and its dynamics would be as described in the last paragraph. Less obviously, an escape mutant that arose on an autosome would also spread. The reason for this is that because it blocks X-cutting it would be transmitted more often to females than its non-escape mutant ‘allele’. When the sex ratio is biased, there is always an advantage to being the rare sex—the mechanism that Fisher recognised underlay the typical 50:50 sex ratio of most organisms. The autosomal escape mutant would thus spread, ultimately restoring an equal sex ratio. For recent modelling of resistance to driving Y chromosomes see Beaghton et al. [23].

## Additional file 1: Note 19. Interacting drives

The advent of CRISPR-Cas9 greatly facilitates the manipulation of DEGs and means that in principle the drive mechanism can be split into different components placed on different chromosomes, and classic population suppression can be combined with Y drive. In a separate file (Additional file 3) we provide a compiled *Mathematica* programme to allow combinatorial exploration of (i) classical suppression and Y drive; (ii) requirement for drive elements on autosomes, Y chromosomes or both; (iii) sex-specific drive; (iv) sex-specific fitness; and (v) variable rates of homing, X chromosome destruction and lethality. Complete exploration of this high-dimensional parameter space is not possible but though many different combinations spread and reduce population fitness, we have failed to find any complex construct that has a clear advantage over simple homing or Y drive strategies (unless the aim is deliberately to design a less efficient DEG). The possibility of independently introducing two DEGs into a population that only exert a fitness cost when carriers of each mate has been raised but not modelled by Esvelt et al. [7].

## Additional file 1: Note 20. Bartonian waves

In cases where there is a threshold DEG density that needs to be exceeded before spread occurs (for example with some forms of heterozygote cost) we would still expect to see a travelling wave, though of a different type called a Bartonian (as opposed to a Fisherian) wave [24]. The most significant difference between the two is that the spread of a Bartonian wave can get stuck at regions of low population densities. The reason for this is that an unproductive habitat may be unable to produce enough migrants to exceed the threshold for spread into an adjacent high-density location. Study of Bartonian waves is particularly important in understanding the spread of *Wolbachia* and overdominant chromosomes [25].

## Additional file 1: Note 21. Heterogeneous landscapes

There is a large literature on the spread of damaging agents (for example, virulent diseases, wildfires) through susceptible landscapes that is relevant to DEG spread [26]. A common result is that as landscapes become more connected a threshold is reached when an introduced gene, disease or wildfire can spread throughout the landscape rather than being confined locally (the threshold is often called the percolation threshold because much of the theory was developed in materials science). Results similar to North et al.’s [27] are also frequently obtained—when densities are low so that random events must be taken into account, local extinctions (in the epidemiology literature often called stochastic fade outs [28]) occur, allowing the affected population to re-establish itself. It is also possible for both host and pathogen to be unable to persist together locally but that neither goes extinct, the interaction persisting by the disease ‘chasing’ the host across the landscape, and the host population recovering when the disease has passed by. It is not yet completely clear the degree to which these dynamics apply to DEGs but their study is important to optimise how any DEGs are deployed in the future.

## Additional file 1: Note 22. No or limited drive endonuclease applications

The capacity of a DEG to spread through a population raises many regulatory issues that will need to be addressed before deployment. It is possible that regulators may require DEGs to be first employed in a more limited way before a decision is made on the release of a completely self-spreading construct.

In their work on Y drive in mosquitoes, Galizi et al. [29], inserted an endonuclease on to an autosome that cuts the X chromosome. Were mosquitoes carrying the construct released into the wild they would cause a sex ratio bias towards males. The autosome carrying the construct would not be specially favoured by the bias (unlike the case where the DEG is on the Y chromosome) and indeed would be disfavoured by being over-represented in the more common sex. They would survive for a limited number of generations and then die out. In principle large numbers of non-driving modified mosquitoes could be released into a population to cause a sex ratio bias, a strategy related to sterile insect release using radiation-treated mosquitoes [30], and the more recent RIDL technique, which involves releasing genetically impaired insects containing self-limiting constructs [31]. However, the most likely use of the autosomal X-cutters is as a staging post to a Y drive strategy.

A further strategy, suggested and modelled by Noble et al. [32], is to insert multiple CRISPR-Cas9 units at different sites in a single genome, such that the target site for each nuclease is opposite the next site in a chain. The unit at the base of the chain will be selected out of the population after a few generations yet elements higher up the chain may be driven to local fixation—so linking the top element to an anti-pathogen gene will enable local dissemination. The system, which Noble et al. call a ‘daisy chain’, is not expected to persist indefinitely in a population yet may be useful for applications where limited DEG spread is desired. Proof of principle of the molecular biology was obtained in a nematode system.

## Additional file 1: Note 23. Tactical models

Most of the results discussed so far have been based on models that are mathematically relatively simple and can be solved to find conditions for spread or genetic and population equilibria, or whose complete behaviour can be explored in relatively low-dimensional parameter space. However, there is a limitation to the amount of biological detail that can be incorporated in such a framework, and more complex simulation models must be used for a more complete description of a particular scenario. Though in principle there is no limit to the detail that can be included, the more complex the model the harder it is to understand its dynamics and to ensure there are no hidden assumptions or errors in the coding.

We are aware of only one case where DEGs have been studied in a biology-rich model, by Eckhoff et al. [13], and this involved the main mosquito vectors of malaria in Africa. The model allows a quite detailed description of the dynamics of larval breeding sites (mosquitoes in the *Anopheles gambiae* group often breed in small puddles whose abundance is strongly influenced by rainfall), competition and predation amongst different larval instars, temperature-specific growth, and behavioural assumptions about how females distribute their eggs. The model keeps track of the absolute numbers of mosquitoes and can be used to study random processes affecting elimination.

Eckhoff et al. studied the spread of a fully recessive DEG with effects only on female fitness in an environment based on well-studied sites in Tanzania and Nigeria, where mosquitoes are common and malaria prevalence high. The climate is highly seasonal (especially in the Nigerian site) and the model linked simulated local weather with the availability of breeding sites and larval development times. Simple theory [5] suggests the DEG should always spread and become fixed when the costs in the female homozygote are above a certain threshold, and that this will lead to population elimination when the load is high enough. In this more realistic model, very weak DEGs sometimes failed to spread because they become extinct through random effects while still rare (an effect that could be countered by more frequent releases). Fixation occurred in a somewhat greater area of parameter space than in the simple models, because of the loss of the wild-type allele by random chance, especially in populations that went through strong seasonal bottlenecks. Importantly, population elimination was predicted for strongly driving DEGs (*e* > ~0.6–0,7) with large fitness effects (*s* > ~0.7), again with seasonal dynamics making this more likely to occur. Similar effects of seasonality and stochasticity were observed with a different type of DEG that biased sex ratios.

The simulations for the Nigerian site were run on a 1×1 km grid as a first step to looking at spatial spread in more realistic landscapes. As expected from general theory, the DEG spreads through the landscape and can collapse the population over large areas. But, especially when local elimination is more frequent due to seasonal bottlenecks, the DEG wavefront can ‘miss’ populations that can subsequently recolonize areas where the vector had been cleared. Such a situation can be countered by more widespread and frequent releases. Finally, the models confirm that resistant mutations can rapidly spread through the population and reverse population suppression.

## Additional file 1: Note 24. Release strategies

The actual deployment of DEGs is still some way off and will rightfully be subject to careful scrutiny and regulation by the appropriate authorities, as well as by civil society. Modelling will be important in designing efficient and cost-effective deployment strategies, as well as in helping to understand rates of spread and establishment for specific constructs targeted at specific pests and vectors. For example, using a spatial model, North et al. [27] looked at the trade-off between releasing a fixed number of insects carrying a DEG at one site versus distributing them in smaller releases across many sites. Because the relationship between establishment probability and the size of release quickly plateaus, multiple releases are usually better to increase the rate of spread of the gene through the environment.

# References

1. Gantz VM, Bier E. The mutagenic chain reaction: a method for converting heterozygous to homozygous mutations. Science. 2015;348(6233):442–4.

2. Gantz VM, Jasinskiene N, Tatarenkova O, Fazekas A, Macias VM, Bier E, et al. Highly efficient Cas9-mediated gene drive for population modification of the malaria vector mosquito *Anopheles stephensi*. Proc Natl Acad Sci U S A. 2015;112(49):E6736–E43.

3. Hammond A, Galizi R, Kyrou K, Simoni A, Siniscalchi C, Katsanos D, et al. A CRISPR-Cas9 gene drive system-targeting female reproduction in the malaria mosquito vector Anopheles gambiae. Nat Biotechnol. 2016;34(1):78–83.

4. Marshall JM, Buchman A, Sánchez HM, Akbari OS. Overcoming evolved resistance to population-suppressing homing-based gene drives. Sci Rep-Uk. 2016;in press.

5. Deredec A, Burt A, Godfray HCJ. The population genetics of using homing endonuclease genes in vector and pest management. Genetics. 2008;179(4):2013–26.

6. Deredec A, Godfray HCJ, Burt A. Requirements for effective malaria control with homing endonuclease genes. Proc Natl Acad Sci U S A. 2011;108(43):E874–E80.

7. Esvelt KM, Smidler AL, Catteruccia F, Church GM. Concerning RNA-guided gene drives for the alteration of wild populations. Elife. 2014;3.

8. Unckless RL, Clark AG, Messer PW. Evolution of resistance against CRISPR/Cas9 gene drive. Genetics. 2017;in press.

9. Gilbert LA, Larson MH, Morsut L, Liu ZR, Brar GA, Torres SE, et al. CRISPR-mediated modular RNA-guided regulation of transcription in eukaryotes. Cell. 2013;154(2):442–51.

10. Sinkins SP, Gould F. Gene drive systems for insect disease vectors. Nat Rev Genet. 2006;7(6):427–35.

11. Unckless RL, Messer PW, Connallon T, Clark AG. Modeling the manipulation of natural populations by the mutagenic chain reaction. Genetics. 2015;201(2):425–31.

12. Noble C, Olejarz J, Esvelt KM, Church GM, Nowak MA. Evolutionary dynamics of CRISPR gene drives. bioRxiv. 2016.

13. Eckhoff PA, Wenger EA, Godfray HCJ, Burt A. Impact of mosquito gene drive on malaria elimination in a computational model with explicit spatial and temporal dynamics. Proc Natl Acad Sci U S A. 2017;114(2):E255–E64.

14. Lande R, Engen S, Saether BE. Stochastic population dynamics in ecology and conservation. Oxford, UK: Oxford University Press; 2003.

15. Courchamp F, Berec L, Gascoigne J. Allee effects in ecology and conservation. Oxford, UK: Oxford University Press; 2008.

16. Goodsman DW, Koch D, Whitehouse C, Evenden ML, Cooke BJ, Lewis MA. Aggregation and a strong Allee effect in a cooperative outbreak insect. Ecol Appl. 2016;26(8):2621–34.

17. David AS, Kaser JM, Morey AC, Roth AM, Andow DA. Release of genetically engineered insects: a framework to identify potential ecological effects. Ecol Evol. 2013;3(11):4000–15.

18. Anderson RM, May RM. Infectious diseases of humans. Oxford University Press: Oxford; 1991.

19. Brady OJ, Godfray HCJ, Tatem AJ, Gething PW, Cohen JM, McKenzie FE, et al. Adult vector control, mosquito ecology and malaria transmission. Int Health. 2015;7(2):121–9.

20. Brady OJ, Godfray HCJ, Tatem AJ, Gething PW, Cohen JM, McKenzie FE, et al. Vectorial capacity and vector control: reconsidering sensitivity to parameters for malaria elimination. Trans R Soc Tropical Med Hygiene. 2016;110(2):107–17.

21. Beaghton A, Hammond A, Nolan T, Crisanti A, Godfray HCJ, Burt A. Requirements for driving anti-pathogen effector genes into populations of disease vectors by homing. Genetics. 2017;205:1587–96.

22. Wu B, Luo LQ, Gao XJJ. Cas9-triggered chain ablation of cas9 as a gene drive brake. Nat Biotechnol. 2016;34(2):137–8.

23. Beaghton A, Beaghton PJ, Burt A. Vector control with driving Y chromosomes: modelling the evolution of resistance. Malaria J. 2017;16:286.

24. Barton NH. The dynamics of hybrid zones. Heredity. 1979;43:341–59.

25. Turelli M, Hoffmann AA. Rapid spread of an inherited incompatibility factor in California *Drosophila*. Nature. 1991;353:440–2.

26. Tilman D, Kareiva P, editors. Spatial ecology: the role of space in population dynamics and interspecific interactions. Princeton, USA: Princeton; 1997.

27. North A, Burt A, Godfray HCJ. Modelling the spatial spread of a homing endonuclease gene in a mosquito population. J Appl Ecol. 2013;50(5):1216–25.

28. Bolker B, Grenfell B. Space, persistence, and dynamics of measle epidemics. Philos Trans R Soc B. 1995;348(1325):309–20.

29. Galizi R, Doyle LA, Menichelli M, Bernardini F, Deredec A, Burt A, et al. A synthetic sex ratio distortion system for the control of the human malaria mosquito. Nat Commun. 2014;5.

30. Knipling EF. Possibilities of insect control or eradication through the use of sexually sterile males. J Economic Entomol. 1955;48(4):459–62.

31. Thomas DD, Donnelly CA, Wood RJ, Alphey LS. Insect population control using a dominant, repressible, lethal genetic system. Science. 2000;287(5462):2474–6.

32. Noble C, Min J, Olejarz J, Buchthal J, Chavez A, Smidler AL, et al. Daisy-chain gene drives for the alteration of local populations. Sci Adv. 2016;3:e1601964.

# Software

As further additional material we provide compiled computer programmes that allow classic homing gene drive and driving Y chromosomes to be explored separately and together (see Additional file 1: Note 19, and Additional file 3) and that allows further exploration of the CATCHA mechanism (see Additional file 1: Note 16, and Additional file 2). The programmes are written in *Mathematica* and can be run using free software (cdf player) that can be downloaded from the Wolfram Mathematica site (<https://www.wolfram.com/cdf-player/>).
